# A joint spatial econometric model for regional FDI and output growth

**DOI:** 10.1111/pirs.12714

**Published:** 2023-02-21

**Authors:** Tamás Krisztin, Philipp Piribauer

**Affiliations:** ^1^ International Institute for Applied Systems Analysis (IIASA) Laxenburg Austria; ^2^ Paris Lodron University of Salzburg (PLUS) Salzburg Austria; ^3^ Austrian Institute of Economic Research (WIFO) Vienna Austria

**Keywords:** European regions, FDI–output relationship, spatial econometric estimation, spatial spillovers

## Abstract

This paper studies the joint dynamics of foreign direct investments (FDI) and output growth in European regions by using spatially augmented systems of equations modeling framework that incorporates third‐region and spillover effects. The joint framework is used to study the dynamic impacts of regional human capital endowments, which demonstrates the importance of explicitly accounting for an endogenous relationship. The relationship is highlighted in a stylized projection exercise, where the long‐run impacts are pronounced in Eastern Europe and capital cities. Overall, ignoring the relationship of regional economic performance and FDI distorts the implied transmission mechanism, which is of utmost importance for policy makers.

## Resumen

Este artículo estudia la dinámica conjunta de la inversión extranjera directa (IED) y el crecimiento de la producción en las regiones europeas utilizando un marco de modelización de sistemas de ecuaciones aumentados espacialmente que incorpora los efectos de tercera región y de *spillover*. El marco conjunto se utiliza para estudiar los efectos dinámicos de las dotaciones regionales de capital humano, lo que demuestra la importancia de tener en cuenta explícitamente una relación endógena. La relación se pone de relieve en un ejercicio de proyección estilizada, en el que los efectos a largo plazo son pronunciados en Europa del Este y en las capitales. En general, ignorar la relación entre los resultados económicos regionales y la IED distorsiona el mecanismo de transmisión implícito, que es de suma importancia para los formuladores de políticas.

## 抄録

本稿では、第三地域とスピルオーバー効果を組み込む空間的に拡張された方程式モデリングのフレームワークを用いて、欧州地域における外国直接投資(FDI)と生産増加の共同ダイナミクスを検討する。この共同フレームワークは、地域の人的資本の動的な影響を研究するために使用されるが、これは内生的な関係を明示的に説明することの重要性を明らかにするものである。この関係は様式化された投影演習において明示されており、また長期的な影響は東欧や首都において顕著であることも指摘される。全体として、地域の経済パフォーマンスとFDIの関係を無視することは、暗示的な伝達のメカニズムを歪めることになるが、これは政策立案者にとって極めて重大なことである。

## 1 INTRODUCTION

There is a vast literature on the determinants of (regional) economic growth both in theoretical and empirical terms. Among numerous other factors, foreign direct investment (FDI) activities play a key role in economic development. From a theoretical perspective, there are several channels through which FDI might foster economic development. Early growth models by Solow (1956) and Swan (1956) emphasize the role of FDI as a driver of investments, while endogenous growth theories highlight the importance of technology and knowledge diffusion processes (Lucas, 1988; Romer, 1986).

At the national level, the role of FDI as a driver of economic growth is empirically well documented (see, e.g., Blonigen et al., 2007; Blonigen & Piger, 2014; Leibrecht & Riedl, 2014; Regelink & Elhorst, 2015, or; Huber et al., 2019). However, studies in a regional context are particularly scarce. When moving from a national to a regional perspective, several issues emerge. The foremost problem concerns data quality, as official data on FDI investments are not readily available. Another challenge concerns the adequate modeling strategies to capture spatial spillover effects.

The importance of FDI for economic development has led to a large body of literature dealing specifically with the determinants of FDI particularly at the national level. However, there are only few studies on the (European) regional level. To circumvent the lack of official data on regional FDI flows, recent papers increasingly rely on alternative data sources such as media reports. Recent examples include Crescenzi et al. (2013), Ascani et al. (2016a), or Krisztin and Piribauer (2020), who make use of data on FDI activities provided by the *fDi Markets* database. This data set uses press information to track the locational decisions of multinational enterprises on a subnational scale.

A key insight of both strands of literature is that, on the one hand, FDI inflows are arguably a key driver for economic development and, on the other hand, economic output is considered a fundamental determinant for attracting FDI inflows. These results therefore suggest a non‐negligible endogenous interrelationship between economic output and FDI, a result that is underpinned not only in a national but also in a regional context. However, only few studies focus on explaining both quantities of interest in isolation and ignore the interdependencies between them. A notable exception in the national context is Huber et al. (2019), showing significant evidence for the presence of links between FDI and economic output. However, in a regional context there is a clear gap of empirical work examining whether such linkages play a significant role on a subnational scale.

This paper provides empirical evidence on the spatial interrelationship between inward FDI and economic growth in European NUTS‐2 regions. For this purpose, a spatially augmented two‐equation modeling framework is developed, which captures spatial spillovers and third‐region effects. The joint framework allows to explicitly dissect the mutual contribution of the FDI–output relationship. First, the paper studies the in‐sample dynamics of the systems of equations modeling framework in European subnational units. Second, the paper provides an analysis of the spatial growth contributions of regional FDI inflows using stylized counterfactual scenario analyses. Specifically, a projection exercise studies the long‐run spatial economic implications when assuming a sharp decline in future FDI due to increased backshoring. The study moreover shows the implications of using a joint modeling framework versus assuming no endogenous link between regional FDI and economic output by comparing the dynamic impacts of regional human capital endowments. From a methodological perspective, the Bayesian estimation approach is related to a recent work on endogenous (spatial) relationships between regional human capital endowments and economic growth (see Crespo Cuaresma et al., 2018). A thorough sensitivity analysis underpins the robustness of the results. The paper therefore adds to the existing literature along several dimensions. To the best of our knowledge, this is the first paper that provides empirical evidence on the impact and spillover effects of regional FDI on output growth in a pan‐European context. The paper highlights the importance of the interdependent spatial relationship between economic output and FDI by explicitly accounting for space‐time dynamics for both quantities. Most importantly, the results show that a separate consideration of the two (endogenous) variables leads to erroneous estimates of the transmission mechanisms, which is of particular relevance for decision makers in economic policy.

The rest of the paper is structured as follows. Section 2 briefly reviews the existing literature on the relationship between FDI and economic growth. Section 3 outlines the employed joint model for economic growth and FDI in a pan‐European regional framework. Section 4 presents the spatial scale and discusses the data sources. In‐sample estimation results for the regional FDI and economic output are discussed in Section 5. Section 6 studies the outcomes of the endogenous empirical setup comparing projected results of stylized scenarios using a counterfactual impact analysis. Section 7 presents empirical sensitivity of our results along several dimensions. The final section concludes.

## 2 THE LITERATURE ON THE RELATIONSHIP BETWEEN (REGIONAL) FDI AND OUTPUT GROWTH

The importance of FDI on economic development has led to a great effort of researchers studying the economic impacts and determinants of FDI activities. Empirical evidence on the impacts of FDI on economic development is, however, controversial (see, e.g., de Mello, 1999). Numerous macroeconomic papers aim at explaining the impacts of FDI activities on economic output. For example, Balasubramanyam et al. (1996) argue that trade‐openness appears to be crucial, to reap the potential growth effects of FDI activities. Early work by Borensztein et al. (1998) moreover emphasizes the importance of a well‐developed human capital stock to benefit from FDI activities. Further growth‐enhancing factors include the degree of embeddedness of foreign firms in local economies (Markusen & Venables, 2000; Rodriguez‐Clare, 1996) or the general business environment (Blomstrom & Kokko, 2003; Xu, 2000). However, most studies do not fully control for third‐country effects or dynamic aspects of the FDI–growth relationship. A notable exception is a recent work by Huber et al. (2017), who use a global vector autoregressive model to analyze the FDI–growth nexus of US FDI activities.

Apart from studying the impacts of FDI on economic growth, several studies aim to analyze the economic determinants of attracting FDI inflows (for a good overview, see, e.g., Blonigen & Piger, 2014, or; Eicher et al., 2012). For theoretical underpinnings on the economic determinants of FDI, the well‐known OLI (ownership‐localization‐internalization) theory (Dunning, 2001) is often used. This theoretical framework aims at explaining the motivation of firms to become multinational by combining both micro‐ and macro‐oriented fundamentals (see also Iammarino & McCann, 2013). Further prominent approaches build on general equilibrium frameworks by Markusen (1984) and Helpman (1984).

Another strand of the literature builds on trade theory to derive empirical specifications from bilateral gravity approaches (for an overview, see Blonigen, 2005). An influential example is the work by Blonigen et al. (2007), which analyzes the determinants of US outbound FDI activities in a cross‐country framework. Some studies also focus on geographic spatial issues for understanding bilateral FDI activities (see Baltagi et al., 2007; Ekholm et al., 2007, or; Hirsch et al., 2020). Recent applications of spatially augmented gravity approaches using European multilateral FDI data, which explicitly incorporate spatial interdependencies, are given by Leibrecht and Riedl (2014) or Krisztin and Piribauer (2020). However, it is worth noting that the vast majority of the empirical literature focuses on country‐specific FDI patterns due to the scarcity of data on a subnational scale. Especially for European regions, most studies look at only a single country or a rather limited time period (among several others, see, e.g., Barrios et al., 2006; Crozet et al., 2004; Devereux et al., 2007; Fallon & Cook, 2010; Guimaraes et al., 2000; or; Casi & Resmini, 2014).

Recently, the widespread availability of alternative data sources such as media reports has led to a substantial increase in empirical studies aimed at analyzing regional FDI patterns. In this context, some articles specifically address the question whether there are sectoral differences in the determinants of FDI attraction. The sectoral distinction increasingly focuses on the idea that the classification is less concerned with the sector as such, but rather with its functional form. Recent studies for European regions include Ascani et al. (2016a), Crescenzi et al. (2013), Duboz et al. (2019), and Krisztin and Piribauer (2020). In the European regional context, several studies have moreover emphasized the role of FDI as an engine of the integration process in neighboring countries (Ascani et al., 2016a; 2016b). In addition to the transmission of essential capital, (European) FDI fosters the diffusion of knowledge and innovation into the European neighborhood (Crescenzi & Petrakos, 2016). Most of these studies make use of data on FDI activities provided by the *fDi Markets* database. This data set uses press information to track the locational decisions of multinational enterprises on a subnational scale.

From a (European) regional perspective, empirical literature on the overall driving forces of economic growth appears much more vast. Recent empirical studies highlight the importance of accounting for spatial autocorrelation using spatial econometric methods (LeSage & Pace, 2009). Motivated by theoretical underpinnings from regional growth theory (see, e.g., Fischer, 2011), these papers typically rely on spatially augmented Barro‐type regressions (Barro, 1991; LeSage & Fischer, 2009) to unveil the determinants of economic growth and associated spatial spillover processes. Recent examples include, among several others, (Crespo Cuaresma et al., 2014), Fischer and LeSage (2015), Piribauer (2016), or Crespo Cuaresma et al. (2018).

However, empirical literature on the impacts of FDI on regional growth appears particularly scarce. A notable exception is a recent work by Gutiérrez‐Portilla et al. (2019), which highlights the effect of FDI inflows on regional economic growth in Spanish regions using spatial econometric methods. The results suggest a pronounced positive (but temporarily delayed) direct impact of FDI on output growth. Interestingly, their results moreover show that the positive spillover effects appear even stronger than the direct impacts. To the best of our knowledge, and also in line with the conclusion in Gutiérrez‐Portilla et al. (2019), the vast majority of studies analyzing the subnational spatial FDI–growth relationship center on Chinese regions (see, e.g., Ma & Jia, 2015; Mitze & Özyurt, 2014; or; Wen, 2014).

## 3 A JOINT MODEL FOR REGIONAL ECONOMIC GROWTH AND FDI

Based on the short literature review, consider a joint spatial panel modeling framework of FDI inflows and economic output for European regions. Let $y_{i,t}$ denote the (log‐level) of per capita gross value added (GVA) in region i at time t (with i=1,…,N and t=1,…,T). Similarly, let $f_{i,t}$ be the stock of inward FDI investments. The empirical modeling framework can be written as follows:

(3.1) $$\text{GVA:}\;y_{t+h}=\rho_{y}Wy_{t+h}+\alpha_{y}+X_{t}^{\left(y\right)}\beta_{y}+WX_{t}^{\left(y\right)}\theta_{y}+\varepsilon_{y},$$

(3.2) $$\text{FDI:}\;f_{t+h}=\psi y_{t+h}+\rho_{f}Wf_{t+h}+\alpha_{f}+X_{t}^{\left(f\right)}\beta_{f}+WX_{t}^{\left(f\right)}\theta_{f}+\varepsilon_{f},$$

where $y_{t}={\left[y_{1,t}\;,\ldots ,y_{N,t}\right]}^{\prime },\;f_{t}={\left[f_{1,t}\;,\ldots ,f_{N,t}\right]}^{\prime }$, and h denotes the lag length. $X_{t}^{\left(m\right)}$ (with m∈[y,f]) are $N\times Q_{m}$ matrices whose rows $x_{i,t}^{\left(m\right)}$ ( $1\times Q_{m}$) are region‐specific data of explanatory variables measured at period t, with equation‐specific parameter vectors $\beta_{m}$. Following standard assumptions and notation in the spatial econometrics literature, matrix W is an exogenously given row‐stochastic N×N spatial weight matrix with non‐negative elements (for a thorough introduction, see LeSage & Pace, 2009). Specifically, element $w_{i,j}>0$ if region i≠j is assumed as a neighbor to region i, and zero otherwise. By construction, $w_{i,i}=0$. The (scalar) parameters $\rho_{m}$ denote spatial autoregressive parameters with sufficient stability condition $\rho_{m}\in (-1,1)$. The parameter vectors $\theta_{m}$ denote column vectors corresponding to spatially lagged explanatory variables. After controlling for initial conditions and spatial spillovers, the nuisance terms $\varepsilon_{m}$ are assumed to be contemporaneously uncorrelated and homoscedastic, $\varepsilon_{m}∼N(0,\sigma_{m}^{2}I_{N})$.

The equations contain spatial lags in both the endogenous and exogenous variables using an N×N spatial weight matrix W and thus constitute well‐known and flexible spatial Durbin specifications (see (LeSage & Pace, 2009)). These spatially augmented regression frameworks have been recently used in the empirical literature on both regional economic growth (see, e.g., Crespo Cuaresma et al., 2014; LeSage & Fischer, 2009; Piribauer, 2016) and FDIs (see, e.g., Baltagi et al., 2007, Blonigen et al., 2007; Krisztin & Piribauer, 2020; Regelink & Elhorst, 2015).

It is, however, important to note that the equation‐specific design matrices $X_{t}^{\left(m\right)}$ also contain both endogenous variables measured at time t ( $y_{t}$ and $f_{t}$, respectively). By conditioning on the starting values, both equations thus represent specific forms of (spatially augmented) Barro‐type (Barro, 1991) growth regressions.

While equation (3.1) represents the equation for regional output, equation (3.2) describes the regional trajectories for FDI stocks. Similar to the economic output equation, the FDI equation also allows for local and global spillovers among the regions. In line with recent literature on the determinants of (regional) FDI, among other potential driving factors, the empirical study explicitly accounts for third‐region effects, the industry mix, and prior FDI inflows. Recent literature particularly identifies the market size (usually proxied by economic output) as one of the most important pull‐factors for FDI. Our FDI equation (3.2) thus also contains a contemporaneous relation to economic output ( $y_{t+h}$) with corresponding parameter ψ.

However, the recursive system of equations modeling framework rests on an important identification assumption. While FDI stocks in equation (3.2) may be contemporaneously affected by output trajectories, regional GVA is only assumed to react sluggishly to FDI announcements. 2 The validity of this assumption was carefully checked from an empirical point of view. Specifically, adding a contemporaneous FDI variable to the GVA equation results in a highly insignificant corresponding parameter, 3 which corroborates our assumption that regional output reacts sluggishly to FDI announcements.

Following recent work on the relationship between economic output and human capital by Crespo Cuaresma et al. (2018), it is useful to rewrite the econometric specifications sketched above into a system of equations. Therefore, define a 2×1 vector $z_{i,t+h}={\left(y_{i,t+h},f_{i,t+h}\right)}^{\prime }$, which collects the two endogenous variables for region i:

(3.3) $$z_{i,t+h}=\Psi^{-1}\left(\alpha +\Phi z_{i,t+h}^{*}+Bx_{i,t}^{\prime }+\Theta x_{i,t}^{*}+e\right),$$

where the matrices $\alpha =\left[\begin{array}{c} \alpha_{y}\\ \alpha_{f} \end{array}\right],\;B=\left[\begin{array}{c} \beta_{y}^{\prime }\\ \beta_{f}^{\prime } \end{array}\right],\;\Theta =\left[\begin{array}{c} \theta_{y}^{\prime }\\ \theta_{f}^{\prime } \end{array}\right]$, and $e=\left[\begin{array}{c} \varepsilon_{y}^{\prime }\\ \varepsilon_{f}^{\prime } \end{array}\right]$ are obtained by vertically stacking the respective equation‐specific quantities in (3.1) and (3.2). Moreover, define:

(3.4) $$\Psi =\left[\begin{array}{cc} 1 & 0\\ -\psi & 1 \end{array}\right],\Phi =\left[\begin{array}{cc} \rho_{y} & 0\\ 0 & \rho_{f} \end{array}\right],z_{i,t+h}^{*}=\left[\begin{array}{c} \sum_{j=1}^{N}w_{i,j}y_{j,t+h}\\ \sum_{j=1}^{N}w_{i,j}f_{j,t+h} \end{array}\right],x_{i,t}^{*}=\left[\begin{array}{c} \sum_{j=1}^{N}w_{i,j}x_{j,t}^{\left(y\right)}\\ \sum_{j=1}^{N}w_{i,j}x_{j,t}^{\left(f\right)} \end{array}\right],$$

where asterisks denote spatially lagged quantities. As the terms on the right‐hand side of the reduced form equation (3.3) only contain exogenous variables in the beginning of the sampling period, the resulting framework is suitable to perform a scenario analysis to study the complex relationship between FDI and economic growth. As the proposed estimation framework has a recursive structure, equations (3.1) and (3.2) can be estimated separately. It is moreover worth noting that in the reduced form of the model, the resulting nuisance term $\Psi^{-1}e∼N(0,\Omega )$ exhibits a contemporaneous correlation in the shocks with variance–covariance matrix given by:

(3.5) $$\Omega =\left(\Psi^{-1}\right)\left(\begin{array}{cc} \sigma_{y}^{2} & 0\\ 0 & \sigma_{f}^{2} \end{array}\right){\left(\Psi^{-1}\right)}^{\prime }.$$

## 4 REGIONS AND SPATIAL DATA

The empirical application uses information on N=258 European NUTS‐2 regions in an annual period from 2006 to 2018 ( T=11), with a growth horizon of 2 years ( h=2). The complete list of regions is presented in Table A1 in the Appendix. An overview of the variables used is given in Table 1. The data are openly available and stem from the Eurostat and ARDECO 4 regional databases. The sole exception is the information on regional FDI activities, which comes from the *fDi Markets* database (maintained by *fDi Intelligence*—a specialist division of the Financial Times Ltd.) and uses information from media sources and company data. Specifically, reported FDI activities in the database contain all cross‐border greenfield investments, and the inclusion of investments in the database is conditional on the FDI flow generating new employment or capital investments in the host region.

In line with recent literature, FDI stocks rather than flows are used because the former are less volatile and thus arguably more suitable to analyze longer‐term effects of FDI (see, among others, Bitzer & Görg, 2009; Ford et al., 2008; or; Kannen, 2020). To transform FDI flows into stocks, the well‐known perpetual inventory method with a depreciation rate of 10*%* was used (Bitzer & Görg, 2009; Sapkota & Bastola, 2017; Wacker, 2011). 5 The paper uses the capital investment variable (*capex*) from the *fDi Markets* database (originally expressed in millions of US dollars), deflated to Euro in 2010 using exchange rates and the national gross domestic product price index from Eurostat as an input for the construction of FDI stocks. In the regional economic literature, the reported *capex* variable has been widely used and is considered as an acceptable proxy for regional FDI connectivity (Burger et al., 2013; Crescenzi & Iammarino, 2017; Iammarino, 2018). 6

Similar to the FDI stocks, the perpetual inventory method with a depreciation rate of 10*%* is used to construct regional knowledge capital stocks. For knowledge capital, this approach has been widely used in the following recent empirical regional economic literature: Fischer and LeSage (2015), LeSage and Fischer (2012), and Krisztin and Piribauer (2020).

**TABLE 1:** Variables in the sample.

Variable	Description
GVA	Regional per‐capital gross value added (constant 2010 Euros), in log terms. *Source*: ARDECO
FDI	FDI stocks (constant 2010 Euros), in log terms. *Source*: *fDi Markets*
Employment in industry	Share of NACE B to F (industry and construction) in total employment. *Source*: ARDECO
Employment in services	Share of NACE G to U (services) in total employment. *Source*: ARDECO
Population density	Total population per square km, in log terms. *Source*: ARDECO
Tertiary education workers	Share of population (aged 25 and above) with higher education (ISCED levels 6+). *Source*: Eurostat
Lower education workers	Share of population (aged 25 and above) with lower education (ISCED levels 0–2). *Source*: Eurostat
Regional knowledge capital	Knowledge stock formation measured in terms of patent accumulation, in log terms. *Source*: Eurostat

*Notes*: ISCED and NACE refer to the international standard classification of education and the second revision of the statistical classification of economic activities in the European community, respectively.

For the spatial weight matrix W, a row‐standardized k‐nearest neighbor specification with k=7 is used. 7 The k‐nearest specification is rather popular in the empirical literature because it has several advantages of contiguity based or other distance‐based metrics particularly in the presence of islands or regions of different size. Several model runs using different values of k as alternative neighborhood structures (see Table 4) underpin the robustness of the results. Robustness was also checked using different Ws for the two equations. Similarly, the results appeared qualitatively rather insensitive to these choices.

## 5 ESTIMATION RESULTS

Estimation is carried out using Bayesian Markov‐chain Monte Carlo (MCMC) techniques for spatial autoregressive models. The main advantage of Bayesian simulation methods for our framework is that it directly produces the entire posterior distribution of the parameters under scrutiny. This appears particularly useful because spatial impact metrics and also the employed scenario designs require the evaluation of nonlinear functions of the unknown parameters, which are severely facilitated when using Bayesian MCMC estimation. For estimation, rather non‐informative and standard prior distributions for the unknown parameters are elicited. Specifically, for the spatial autoregressive parameters a uniform (flat) prior U(−1,1) is used. For the slope coefficients and the error variances, normal priors $N(0,10^{2}I)$ with large prior uncertainty and an inverted gamma distribution $IG(10^{-3},10^{-3})$ with diffuse settings are used, respectively. The prior setup can thus be viewed rather non‐informative and is commonly used in the (spatial) econometric literature (see, e.g., LeSage & Pace, 2009.)

Table 2 presents the posterior results for both equations based on 3000 posterior draws after discarding the first 2000 as burn‐ins. 8 It is worth noting that due to the nonlinear nature of spatial autoregressive models, slope parameter estimates do not represent partial derivatives. The paper therefore follows common practice in the literature and reports spatial summary metrics in the form of average direct and indirect (spillover) impacts proposed by LeSage and Pace (2009).

Average direct effects denote the average impact of the dependent variable in region i due to a marginal increase in a particular explanatory variable in the same region. Direct impacts are thus reminiscent of slope parameter estimates in classical linear models. Average indirect (or spillover) effects measure the average impact on the dependent variable in region i due to a joint marginal increase in an explanatory variable in all regions other than region i. As an alternative (but equivalent) interpretation, average indirect (spillover) effects can also be represented as the average (cumulative) impact of the dependent variable in all regions other than i due to a marginal shock in region i. 9

The table depicts posterior mean estimates and standard deviations for the summary impact metrics for both equations along with estimates for the spatial autoregressive and nuisance variance parameters. Impact metrics and parameter estimates significant under a 90*%* posterior credible interval are highlighted in bold. 10 The results for the growth equation (depicted in the upper part of Table 2) show a rather strong (and highly significant) degree of spatial autocorrelation (0.85). Most importantly, the table also shows that the FDI stock variable positively affects per capita income growth. 11 Table 2 shows direct and indirect spillover impact estimates that appear both positive and significant. For the remaining explanatory variables, the results are generally in line with recent empirical literature on European regional growth determinants. Specifically, the results show a particularly pronounced importance of the variables that proxy human capital endowments (tertiary education and lower education workers). Both of their posterior mean estimates for the average direct impacts show the expected signs, with a positive direct impact of tertiary education worker levels and a negative own‐regional impact of the lower education workers variable, respectively. Similar to the work by Olejnik (2008) or Piribauer and Fischer (2015), the indirect (spillover) effects point in the opposite directions. They appear, however, insignificant. The table also suggests positive spillover effects of knowledge capital stock. An increase in a region's knowledge stock is thus positively related to per capita output growth in other regions. For the sample under scrutiny, the results suggest negative direct impacts of the share in market services (measured relative to the agricultural sector), as well as an average direct impact estimate of initial per capita income smaller than unity, suggesting conditional income converging processes.

The results for the regional FDI growth equation are presented in the lower part of Table 2. For the FDI equation, the table shows a markedly weaker but also positive and highly significant degree of spatial autocorrelation. Both (logged) initial and contemporaneous per capita GVA show significant direct impacts on regional FDI stocks. However, it is important to note that the two impact estimates act in opposite directions. Because an increase (decrease) in the log of initial (contemporaneous) economic output ceteris paribus results in a decrease (increase) in the growth rate of FDI stocks, both estimates thus suggest that a ceteris paribus increase in output growth accelerates FDI inflows. This result is closely related to findings in the recent (regional) FDI literature, where proxies for (regional) market potential often appear as the most important drivers for FDI inflows (see Baltagi et al., 2007; Blonigen & Piger, 2014; Krisztin & Piribauer, 2021).

A common result in the empirical literature is also reflected in a positive direct impact estimate for the population density variable (see, e.g., Strauss‐Kahn & Vives, 2009). Moreover, the results also show conditional convergence processes in FDI stocks, which appear even more pronounced compared to those for economic output. Own‐regional knowledge endowments as well as knowledge spillovers both appear of particular importance as a means to attracting FDI inflows. Concerning the industry mix variables, Table 2 suggests positive direct impacts of higher share of industry as well as positive spillover effects from a higher share in services. However, there are no significant direct or indirect impacts for the human capital variables. This result is also in line with recent literature since educational attainment levels are often claimed to be of particular importance for attracting only certain functional types of FDI (Crescenzi et al., 2013; Defever, 2006; Fallon & Cook, 2014).

**TABLE 2:** Baseline results.

	Direct		Indirect	
	Mean	SD	Mean	SD
	Growth Equation			
GVA 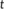	**0.9872**	**0.0092**	−0.0127	0.1954
FDI 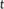	**0.0037**	**0.0006**	**0.0180**	**0.0077**
Employment in industry	−0.0036	0.0199	−0.1448	0.1912
Employment in services	−**0.0370**	**0.0180**	−0.1594	0.1877
Population density	−0.0003	0.0009	0.0033	0.0073
Tertiary education workers	**0.0423**	**0.0168**	−0.0914	0.1073
Lower education workers	−**0.0784**	**0.0135**	0.0404	0.0500
Regional knowledge capital	−0.0003	0.0003	**0.0067**	**0.0034**
$\rho^{y}$	**0.8494**	**0.0213**		
$\sigma_{y}^{2}$	**0.0010**	**0.0001**		
	FDI Equation			
GVA 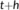	**0.4348**	**0.2072**	−**0.4966**	**0.3715**
GVA 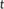	−**0.3463**	**0.2068**	0.1523	0.3648
FDI 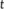	**0.9152**	**0.0058**	0.0310	0.0683
Employment in industry	**0.4406**	**0.1873**	0.0968	0.4057
Employment in services	0.0018	0.1633	0.4348	0.3913
Population density	**0.0342**	**0.0094**	−0.0203	0.0175
Tertiary education workers	0.2055	0.1827	−0.1104	0.2655
Lower education workers	0.1778	0.1480	−0.2053	0.1736
Regional knowledge capital	**0.0081**	**0.0024**	**0.0144**	**0.0066**
$\rho^{f}$	**0.1818**	**0.0414**		
$\sigma_{f}^{2}$	**0.1135**	**0.0030**		

*Notes:* Based on a panel with N=258,T=11, and h=2. Per capita growth specification from 2006 to 2018. Growth‐dependent variable is log levels per capita GVA. FDI‐dependent variable is log of FDI investment stock. GVA 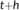 denotes the contemporaneous output variable, while GVA 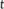 represents the initial level. Figures in bold indicate significance under a 90*%* posterior credible interval.

## 6 COUNTERFACTUAL IMPACT ANALYSIS

While the previous section has analyzed the in‐sample properties and determinants of both regional quantities under scrutiny, the joint modeling framework appears particularly useful for a counterfactual impact analysis. Therefore, consider two alternative scenarios. First, the paper analyzes the expected impacts of a (positive) human capital shock on both endogenous variables. The expected impacts of the joint model are then contrasted to the results when assuming no endogenous link between regional FDI and economic output. Second, the paper studies the spatial growth premium of FDI inflows using a projection exercise by analyzing the regional expected steady‐state growth paths for economic output (by holding all exogenous factors constant) and contrast it with the model's results when assuming no further FDI inflows. A comparison of the two scenarios thus allows to shed light on the spatial growth premium for economic output resulting from FDI inflows.

### 6.1 The dynamic impacts of a marginal and permanent increase in human capital

This subsection analyzes the model's predictions of a positive human capital shock when using (i) a joint modeling framework (*joint model*) and (ii) when assuming that there is no endogenous link between regional FDI and output (*separate models*). Because the model includes two exogenous variables associated with human capital (lower education workers and tertiary education attainment), define the marginal and positive human capital shock as a one percentage point increase in tertiary education attainment along with a one percentage point decrease in the lower education workers variables at the final period of the sample. By holding all other exogenous variables constant, the dynamic spatial panel setup is used to trace out the expected ceteris paribus average direct and indirect (or spillover) impacts over time (see Debarsy et al., 2012). By keeping the positive human capital shock for all simulated time periods active, the implied average direct and indirect effects thus refer to a permanent (rather than a one‐period) increase in human capital.

**FIGURE 1:**
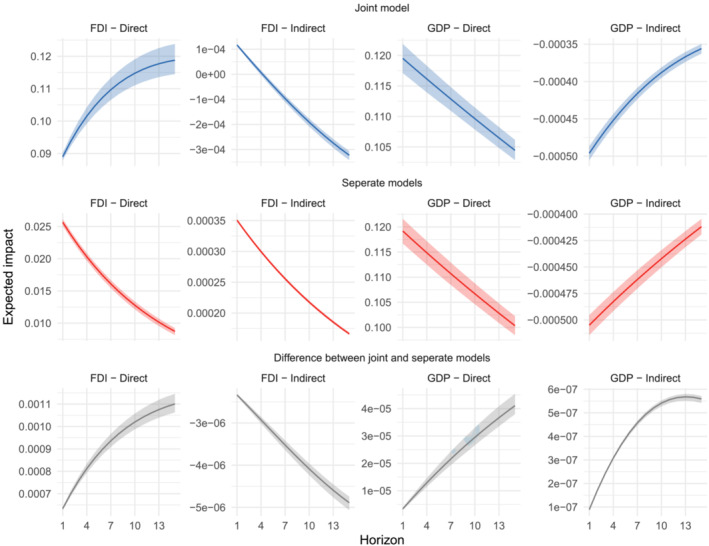
Dynamic impacts of a permanent increase in human capital for the joint model and assuming no endogenous link between economic output and FDI *Notes*: The figure depicts the posterior expectation of the dynamic impacts of a marginal and permanent increase in human capital along with the expected uncertainty over regions for the proposed joint specification (left subplots, blue) vis‐á‐vis to the results when assuming no endogenous link between regional economic output and FDI (right subplots, red), and the difference between blue and red trajectories (third row of subplots, grey). The shaded areas indicate the 16th and the 84th percentiles and indicate the uncertainty of the mean impacts across regions.

To visualize the implications of the joint modeling setup, one may also consider the implied impacts when assuming no endogenous link between regional FDI and economic output. The posterior expected impacts for both scenarios are summarized in Figure 1. The first row of subplots (in blue color) shows average direct and indirect impacts for the *joint model* and the second row of subplots (in red color) those when assuming the standard assumption of no endogenous link (*separate models*). The shaded areas indicate the 16th and the 84th percentiles and depict the uncertainty over regions. For the joint modeling setup, the figure shows marked positive direct (i.e., own regional) impacts of a permanent increase in human capital stocks on FDI inflows. This positive impact on FDI growth is accelerating even further for a longer time horizon. When considering no endogenous feedback effects between FDI and output growth, the dynamic impacts appear much more muted. Moreover, the results suggest that in this case the own regional positive impacts are becoming smaller for longer time periods. The dynamics of the average indirect (or spillover) impacts are generally very similar between the two model frameworks. However, in both cases, the implied indirect impact trajectories appear very small.

When focusing on the impacts of a permanent increase in human capital on output growth, Figure 1 shows rather similar effects as for FDIs. For both model specifications, the direct impacts on output growth are quite pronounced, but decelerate for longer time periods. Overall, the estimated impacts appear slightly larger in the joint setup as compared to those assuming no feedback between FDI and output. When considering the average impact of an own‐regional permanent increase in human capital only on other regions (i.e., the indirect effects), the estimated impacts are negative but less pronounced as compared to the direct effects. For both model variants, the negative spillover impacts on output growth, however, appear to become more muted for longer time periods.

To better visualize the implications of using a joint modeling framework instead of a standard model without any endogenous feedback effects, the third row of Figure 1 depicts the differences between the joint and the standard models. As described before, these plots show marked differences in the dynamic impact assessments especially for the FDI trajectories, while the differences on economic output appear more muted. These results highlight the particular importance of controlling for the endogenous spatial links when studying the spatial behavior of regional FDI.

### 6.2 Dissecting the growth premium of regional FDI inflows: A projection exercise

This subsection studies the complex interdependence between FDI and economic output using an out‐of‐sample projection exercise based on stylized scenarios. For illustration, this subsection focuses on comparing the results of two stylized scenario frameworks on the development of FDI investments. Specifically, the spatial growth contribution of pan‐European regional FDI stocks is analyzed by comparing the future FDI trajectories implied by the joint modeling framework (*baseline scenario*) with the stylized case of fixing FDI stocks to the levels in the end of our sampling period (*no‐investment scenario*). As the no‐investment scenario assumes no further FDI inflows, FDI stocks thus decline over time due to the assumed depreciation rate of 10%. This scenario thus represents the lower limit of future investment trajectories. The no‐investment scenario can be viewed as an extreme case in the context of the recent debate on whether increased efforts toward backshoring are to be expected in the aftermath of the pandemic (see, e.g., Elia et al., 2021).

**FIGURE 2:**
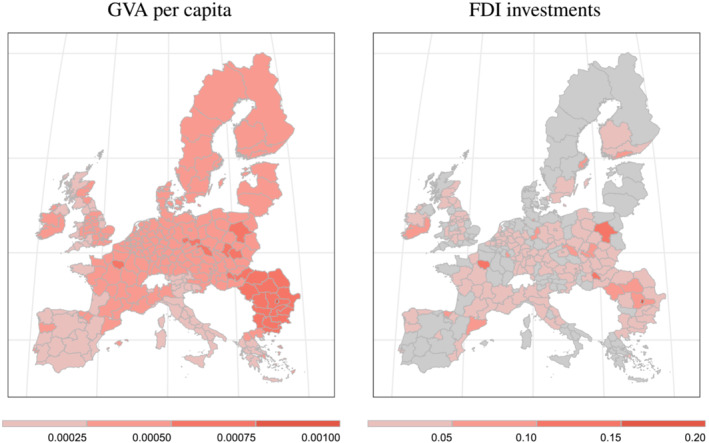
Average annual long‐run log differences between baseline and no‐investment scenarios *Notes*: The maps depict the projected (log difference) surplus of FDI investments (right panel) which then translates to higher GVA per capita growth (left panel) of our model prediction (baseline scenario) vis‐á‐vis a stylized scenario of no further growth in regional FDI (no‐investment scenario)

Using the full sample of MCMC draws, equations (3.2) and (3.1) allow us to produce the predictive densities of out‐of‐sample projections of regional trajectories of FDI stocks and economic output. By iterating the process baseline scenario projections can be produced to obtain paths that extend over a longer prediction horizon. To produce stylized baseline projections, all exogenous variables are held constant.

For both the baseline and the no‐investment scenario implied output impacts are produced for 10 years. Figure 2 depicts average annual log differences in per capita income (left plot) and regional FDI stocks (right plot) between the benchmark and the no‐investment scenario. The right plot in the figure thus reflects the projected annual growth premium, which emanates from European FDI trajectories. Figure 2 projects rather heterogeneous future growth paths of FDI stocks across space. However, growth rates of FDI stock in European capital city regions appear particularly pronounced, especially in central and eastern European (CEE) regions but also regions in central Europe (most notably regions in Germany, France, and Austria). The maps moreover show that both regional (most notably in capital city and metropolitan regions) and country‐specific factors play an important role for attracting FDI. This finding provides an interesting link to the literature, which focuses on the national and regional determinants of FDI inflows (see, e.g., Casi & Resmini, 2014).

The implied effects on regional output growth (compared to the no‐investment case) is depicted in the left plot in Figure 2. This plot similarly shows a particularly pronounced output growth premium in urban and CEE regions and moreover highlights the strong importance of spatial spillover effects. The implied long‐run differences on GVA per capita also reveal the importance of both regional and country‐specific characteristics, where the national factors of FDI on output growth appear particularly pronounced in Bulgaria and Romania.

## 7 ROBUSTNESS OF THE RESULTS

This section discusses the sensitivity of the estimation results based on multiple alternative model specification. These in‐sample robustness checks are presented in Tables 3 and 4. Spatial autoregressive models are based on spatial weight matrices. However, there are alternative ways on how to construct spatial weight matrices and theory typically offers rather limited guidance on the choice of the neighborhood structure (for a thorough discussion, see LeSage & Pace, 2009). Table 4 thus provides the in‐sample results using alternative k‐nearest neighbor structures (baseline results are based on k=7). Specifically, the table shows the spatial impact estimates for k∈{5,10}. Overall, comparing our baseline results in Table 2 with those in Table 4 shows nearly identical spatial impact metrics for both equations under scrutiny.

Robustness checks using alternative model specifications and variable designs are presented in Table 3. The first block labeled as *contemporaneous FDI* relaxes the assumption that per capita GVA reacts only sluggishly to FDI inflows also by including a contemporaneous FDI variable to the output growth equation. The results indeed show no significant direct or indirect (or spillover) impacts of the contemporaneous FDI variable to regional per capita output growth. While the inclusion of contemporaneous FDI also results in insignificant direct impacts of lagged FDI stocks, the estimated spillover effects of lagged FDI remain significant. However, estimated impact metrics of the other (exogenous) variables remain rather robust to the baseline results.

The second block in Table 3 (*Starting period 2010*) presents results when changing the starting period of our panel from 2006 to 2010 to alleviate potential impacts due to the financial crisis. However, a comparison with our baseline results shows only little sensitivity in the estimated impact metrics.

The third block in Table 3 (*Number of FDI inflows*) depicts in‐sample estimation results when choosing an alternative metric to proxy regional FDI stocks. While the baseline results are based on FDI stocks, in this robustness check the (logged) cumulated number of FDI projects to proxy FDI stocks is used. Interestingly, even when using this alternative proxy for FDI stocks, the estimated impact metrics for both the output and FDI equation appears rather robust. While the signs and the precision of the estimated quantities of the core variables appear rather similar, this alternative proxy for FDI exhibits a markedly stronger degree of spatial autocorrelation. In addition to the baseline results and robustness checks summarized in the tables, several additional sensitivity checks have been conducted, which corroborate the robustness of our results. Most notably, robustness checks using alternative lag lengths h have also been conducted. These results as well as the corresponding R codes are available from the authors upon request.

**TABLE 3:** Robustness checks using alternative model specifications.

	Contemporaneous FDI				Starting period 2010				Number of FDI inflows			
	Direct		Indirect		Direct		Indirect		Direct		Indirect	
	Mean	SD	Mean	SD	Mean	SD	Mean	SD	Mean	SD	Mean	SD
	Growth Equation											
GVA 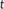	**0.9865**	**0.0092**	−0.0148	0.1961	**0.9809**	**0.0097**	0.0375	0.1860	**0.9836**	**0.0091**	−0.0587	0.1965
FDI 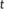	0.0011	0.0023	**0.0382**	**0.0274**	**0.0049**	**0.0007**	**0.0222**	**0.0065**	**0.0052**	**0.0012**	0.0069	0.0101
FDI 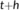	0.0028	0.0023	−0.0222	0.0280								
Employment in industry	−0.0040	0.0195	−0.1335	0.1919	−0.0045	0.0234	−**0.2782**	**0.1481**	0.0103	0.0197	0.1035	0.1759
Employment in services	−**0.0364**	**0.0177**	−0.1630	0.1886	−**0.0679**	**0.0204**	−0.0627	0.1370	−**0.0333**	**0.0179**	−0.0127	0.1876
Population density	−0.0005	0.0009	0.0036	0.0072	0.0009	0.0010	−0.0048	0.0052	0.0001	0.0009	0.0080	0.0074
Tertiary education workers	**0.0422**	**0.0170**	−0.0795	0.1070	**0.0605**	**0.0201**	−**0.1623**	**0.0811**	**0.0487**	**0.0169**	0.0319	0.0949
Lower education workers	−**0.0788**	**0.0134**	0.0410	0.0498	−**0.0665**	**0.0168**	−0.0260	0.0433	−**0.0778**	**0.0133**	**0.0727**	**0.0497**
Regional knowledge capital	−0.0003	0.0003	**0.0070**	**0.0036**	−**0.0006**	**0.0003**	−0.0001	0.0026	0.0000	0.0003	**0.0096**	**0.0036**
$\rho^{y}$	**0.8502**	**0.0212**			**0.7499**	**0.0330**			**0.8552**	**0.0216**		
$\sigma_{y}^{2}$	**0.0010**	**0.0000**			**0.0009**	**0.0000**			**0.0010**	**0.0000**		
	FDI Equation											
GVA 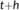	**0.4366**	**0.2004**	−**0.4923**	**0.3712**	**0.4835**	**0.2028**	−0.2380	0.4622	**0.5237**	**0.2533**	−**1.8321**	**1.0561**
GVA 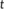	−**0.3479**	**0.2007**	0.1487	0.3669	−**0.3873**	**0.2008**	0.0561	0.4580	−**0.5092**	**0.2526**	**1.4132**	**1.0116**
FDI 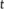	**0.9153**	**0.0056**	0.0310	0.0692	**0.9427**	**0.0059**	−0.0065	0.0834	**0.6258**	**0.0158**	**0.2186**	**0.1089**
Employment in industry	**0.4336**	**0.1891**	0.1082	0.4119	0.1191	0.1927	0.1468	0.4396	**0.6888**	**0.2432**	0.1460	1.0817
Employment in services	−0.0031	0.1670	0.4345	0.3922	−0.1454	0.1626	0.2383	0.4185	0.2539	0.2173	0.2574	1.1544
Population density	**0.0342**	**0.0099**	−0.0196	0.0177	**0.0220**	**0.0090**	−**0.0400**	**0.0183**	**0.0906**	**0.0120**	−**0.1911**	**0.0482**
Tertiary education workers	0.2097	0.1781	−0.1133	0.2679	−0.0843	0.1750	0.1824	0.2758	**0.2826**	**0.2210**	−0.0095	0.6125
Lower education workers	0.1824	0.1437	−0.2084	0.1730	0.0827	0.1502	−**0.3007**	**0.1855**	−**0.2922**	**0.1795**	0.0165	0.3547
Regional knowledge capital	**0.0082**	**0.0023**	**0.0143**	**0.0066**	**0.0055**	**0.0023**	0.0023	0.0072	**0.0147**	**0.0032**	0.0257	0.0203
$\rho^{f}$	**0.1817**	**0.0426**			**0.2503**	**0.0473**			**0.6907**	**0.0249**		
$\sigma_{f}^{2}$	**0.1135**	**0.0031**			**0.0683**	**0.0022**			**0.1834**	**0.0048**		

*Notes: Contemporaneous FDI* additionally includes a contemporaneously FDI stock variable in the output equation (FDI 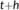). *Starting period 2010* omits the core period of the global financial crisis by choosing a later starting period of the sample (2010–2016, T=7). *Number of FDI inflows* uses the number of regional FDI inflows other than pecuniary investment amounts to proxy FDI stocks. Figures in bold indicate significance under a 90*%* posterior credible interval.

**TABLE 4:** Robustness checks using alternative spatial weight matrices.

	5‐nearest neighbors				10‐nearest neighbors			
	Direct		Indirect		Direct		Indirect	
	Mean	SD	Mean	SD	Mean	SD	Mean	SD
	Growth Equation							
GVA 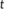	**0.9868**	**0.0095**	−0.0179	0.1418	**0.9871**	**0.0089**	−0.0164	0.2895
FDI 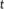	**0.0038**	**0.0006**	**0.0095**	**0.0047**	**0.0036**	**0.0007**	**0.0241**	**0.0132**
Employment in industry	0.0050	0.0198	−0.0773	0.1203	−0.0157	0.0207	−0.0298	0.2930
Employment in services	−**0.0387**	**0.0176**	−0.0446	0.1198	−**0.0461**	**0.0189**	−0.1915	0.2857
Population density	−0.0001	0.0009	−0.0005	0.0047	−0.0001	0.0009	**0.0160**	**0.0117**
Tertiary education workers	**0.0408**	**0.0171**	−0.0349	0.0705	**0.0388**	**0.0168**	−0.0721	0.1645
Lower education workers	−**0.0814**	**0.0137**	0.0131	0.0361	−**0.0726**	**0.0133**	**0.0951**	**0.0691**
Regional knowledge capital	−0.0004	0.0003	**0.0046**	**0.0022**	−0.0002	0.0003	**0.0095**	**0.0059**
$\rho^{y}$	**0.8077**	**0.0206**			**0.8788**	**0.0238**		
$\sigma_{y}^{2}$	**0.0010**	**0.0000**			**0.0010**	**0.0000**		
	FDI Equation							
GVA 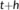	**0.4557**	**0.2043**	−0.3368	0.3176	**0.4434**	**0.2034**	−**0.6560**	**0.4226**
GVA 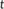	−**0.3584**	**0.2047**	0.0450	0.3153	−**0.3828**	**0.2028**	0.0552	0.4139
FDI 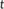	**0.9161**	**0.0055**	0.0223	0.0538	**0.9158**	**0.0056**	−0.0040	0.0842
Employment in industry	**0.3898**	**0.1947**	0.1154	0.3265	**0.4359**	**0.1960**	**1.1924**	**0.4941**
Employment in services	−0.0465	0.1709	**0.4269**	**0.3163**	0.0701	0.1670	**1.1423**	**0.4709**
Population density	**0.0290**	**0.0096**	−0.0100	0.0149	**0.0352**	**0.0094**	−**0.0363**	**0.0216**
Tertiary education workers	**0.2406**	**0.1872**	−0.1351	0.2429	0.1571	0.1704	**0.4130**	**0.3146**
Lower education workers	**0.2425**	**0.1541**	−**0.2898**	**0.1728**	**0.2086**	**0.1426**	−0.1522	0.1799
Regional knowledge capital	**0.0090**	**0.0023**	**0.0068**	**0.0050**	**0.0085**	**0.0023**	**0.0381**	**0.0093**
$\rho^{f}$	**0.0892**	**0.0370**			**0.2411**	**0.0486**		
$\sigma_{f}^{2}$	**0.1143**	**0.0030**			**0.1128**	**0.0031**		

*Notes:* Figures in bold indicate significance under a 90*%* posterior credible interval.

## 8 CONCLUDING REMARKS AND FUTURE RESEARCH

This paper proposes an empirical two‐equation panel modeling framework to study the joint relationship of economic output and FDI stocks in European regions. The empirical framework allows for both global and local spillover mechanisms in both equations and can be estimated using standard Bayesian approaches for spatial autoregressive models. The joint modeling framework sheds light on the complex nexus between regional FDI and output growth and combines official (socio‐)economic data on a pan‐European subnational level with data on greenfield FDI investment activities based on the *fDi Markets* database, which uses information from media sources and company data. In‐sample results highlight the importance of jointly modeling spatial patterns of FDI investments and economic output as well as explicitly capturing spatial spillover processes for both endogenous variables. By studying the dynamic impacts of human capital endowments, the paper moreover shows the importance of the endogenous relationship and sheds light on the spatial growth premium of FDI inflows on regional output growth. Moreover, the results show that studying regional economic performance and FDI in isolation distorts the implied transmission mechanisms, which are of utmost importance for policy makers. Robustness of the results has been checked using a multitude of alternative model specifications.

The paper moreover uses the joint framework in a projection exercise, which allows us to trace out the economic implications of future assumptions on the evolution of key variables. For example, there is a recent debate on whether increased tendencies toward regionalization or backshoring of FDI will emerge in the post‐COVID era (see, e.g., Elia et al., 2021). As an empirical illustration, the paper therefore aims at quantifying the projected long‐run impacts when assuming sharp declines in the future growth of FDI stocks.

However, it is worth noting that the present paper focuses on the spatial relationship between *aggregate* regional FDI and output growth. In line with recent research on the functional role of FDI in the value chain, an interesting avenue for further research is the growth effects emanating from different types of FDI. For this purpose, the approach of recent work by Bolea et al. (2022) appears particularly interesting. Bolea et al. (2022) shed light on the engagement and position of European regions in global value chains and use these metrics to analyze the impact of global production fragmentation on regional economies.

A further limitation of the present spatial econometric analysis is the fact that it focuses on purely geographical spatial weight matrices. While geographical weight matrices are popular due to considerations of exogeneity, the use of alternative sources of information might deepen the understanding on the nature of spillover processes for both subnational output growth and FDI. Recently, Krisztin and Piribauer (2022) provide an approach to estimate unknown spatial weight matrices for relatively large regional panels. Using information on regional production fragmentation, the approach might help to deepen the understanding the complex relationship between regional FDI and economic development.

## Footnotes

2 This assumption is in line with a recent study by Gutiérrez‐Portilla et al. (2019), which also assumes a sluggish reaction of economic output to FDI shocks.

3 Empirical underpinnings for the assumptions are provided in Table 3.

4 ARDECO is the Annual Regional Database of the European Commission's Directorate General for Regional and Urban Policy, maintained and updated by the Joint Research Centre.

5 The first year for the calculation of the capital stock is 2003, the earliest available in the *fDi Markets* database.

6 Several comparisons of the *fDi Markets* data with other (macro‐level) FDI data sets support the reliability and spatial distribution of the FDI data (see, among others, Burger et al., 2013, Crescenzi, 2014, and; Ascani et al., 2016a).

7 Robustness checks using alternative definitions of spatial neighborhood are presented in a separate section.

8 The successful convergence of the MCMC algorithm was carefully checked via the convergence diagnostics proposed by Geweke (1992) and Raftery and Lewis (1992). Convergence diagnostics have been calculated using the R package **coda**.

9 For a detailed and thorough discussion on the interpretation of spatial autoregressive (Durbin) type of models, see LeSage and Pace (2009).

10 The significance of the results is largely unchanged under 95*%* confidence intervals. The only exception is that the indirect impact of the contemporaneous GVA variable in the FDI equation appears insignificant.

11 Overall, this result also appears rather robust to various other modeling frameworks using alternative definitions of FDI stocks, spatial weights, or sampling periods. Details can be found in a separate section. The robustness section also provides an empirical justification on the importance of initial rather than contemporaneous FDI stocks in the GVA equation.

## 1

**TABLE A1:** List of regions in the sample.

Austria	France [continued]	Hungary	Poland [continued]	UK
Burgenland (AT)	Languedoc‐Roussillon	Dél‐Alföld	Lódzkie	Bedfordshire and Hertfordshire
Kärnten	Limousin	Dél‐Dunántúl	Lubelskie	Berkshire, Buckinghamshire and
Niederösterreich	Lorraine	Észak‐Alföld	Lubuskie	Oxfordshire
Oberösterreich	Midi‐Pyrénées	Észak‐Magyarország	Malopolskie	Cheshire
Salzburg	Nord ‐ Pas‐de‐Calais	Közép‐Dunántúl	Mazowieckie	Cornwall and Isles of Scilly
Steiermark	Pays de la Loire	Közép‐Magyarország	Opolskie	Cumbria
Tirol	Picardie	Nyugat‐Dunántúl	Podkarpackie	Derbyshire and Nottinghamshire
Vorarlberg	Poitou‐Charentes	**Ireland**	Podlaskie	Devon
Wien	Provence‐Alpes‐Cte d'Azur	Border, Midland and Western	Pomorskie	Dorset and Somerset
**Belgium**	Rhne‐Alpes	Southern and Eastern	Slaskie	East Anglia
Prov. Antwerpen	**Germany**	**Italy**	Swietokrzyskie	East Wales
Prov. Brabant Wallon	Arnsberg	Abruzzo	Warminsko‐Mazurskie	East Yorkshire and
Prov. Hainaut	Berlin	Basilicata	Wielkopolskie	Northern Lincolnshire
Prov. Liége	Brandenburg	Calabria	Zachodniopomorskie	Eastern Scotland
Prov. Limburg (BE)	Braunschweig	Campania	**Portugal**	Essex
Prov. Luxembourg (BE)	Bremen	Emilia‐Romagna	Alentejo	Gloucestershire, Wiltshire and
Prov. Namur	Chemnitz	Friuli‐Venezia Giulia	Algarve	Bristol
Prov. Oost‐Vlaanderen	Darmstadt	Lazio	rea Metropolitana de Lisboa	Greater Manchester
Prov. Vlaams‐Brabant	Detmold	Liguria	Centro (PT)	Hampshire and Isle of Wight
Prov. West‐Vlaanderen	Dresden	Lombardia	Norte	Herefordshire, Worcestershire
Région de Bruxelles‐Capitale	Düsseldorf	Marche	**Romania**	and Warwickshire
**Bulgaria**	Freiburg	Molise	Bucuresti ‐ Ilfov	Highlands and Islands
Severen tsentralen	Gießen	Piemonte	Centru	Inner London
Severoiztochen	Hamburg	Provincia Autonoma di Bolzano/	Nord‐Est	Kent
Severozapaden	Hannover	Bozen	Nord‐Vest	Lancashire
Yugoiztochen	Karlsruhe	Provincia Autonoma di Trento	Sud ‐ Muntenia	Leicestershire, Rutland and
Yugozapaden	Kassel	Puglia	Sud‐Est	Northamptonshire
Yuzhen tsentralen	Koblenz	Sardegna	Sud‐Vest Oltenia	Lincolnshire
**Czech Republic**	Köln	Sicilia	Vest	Merseyside
Jihováchod	Leipzig	Toscana	**Slovakia**	North Eastern Scotland
Jihozápad	Lüneburg	Umbria	Bratislavská kraj	North Yorkshire
Moravskoslezsko	Mecklenburg‐Vorpommern	Valle d'Aosta/Vallée d'Aoste	Stredné Slovensko	Northern Ireland (UK)
Praha	Mittelfranken	Veneto	Váchodné Slovensko	Northumberland and Tyne and
Severováchod	Münster	**Latvia**	Západné Slovensko	Wear
Severozápad	Niederbayern	Latvija	**Slovenia**	Outer London
Strední Cechy	Oberbayern	**Lithuania**	Vzhodna Slovenija	Shropshire and Staffordshire
Strední Morava	Oberfranken	Lietuva	Zahodna Slovenija	South Western Scotland
**Denmark**	Oberpfalz	**Luxemburg**	**Sweden**	South Yorkshire
Hovedstaden	Rheinhessen‐Pfalz	Luxemburg	Mellersta Norrland	Surrey, East and West Sussex
Midtjylland	Saarland	**Netherlands**	Norra Mellansverige	Tees Valley and Durham
Nordjylland	Sachsen‐Anhalt	Drenthe	Ã–stra Mellansverige	West Midlands
Sjbrvbar;lland	Schleswig‐Holstein	Flevoland	Ã–vre Norrland	West Wales and The Valleys
Syddanmark	Schwaben	Friesland (NL)	Smland med öarna	West Yorkshire
**Estonia**	Stuttgart	Gelderland	Stockholm	
Eesti	Thüringen	Groningen	Sydsverige	
**Finland**	Trier	Limburg (NL)	Västsverige	
Åland	Tübingen	Noord‐Brabant	**Spain**	
Etelä‐Suomi	Unterfranken	Noord‐Holland	Andalucía	
Helsinki‐Uusimaa	Weser‐Ems	Overijssel	Aragón	
Länsi‐Suomi	**Greece**	Utrecht	Cantabria	
Pohjois‐ja Itä‐Suomi	Anatoliki Makedonia, Thraki	Zeeland	Castilla y León	
**France**	Attiki	Zuid‐Holland	Castilla‐la Mancha	
Alsace	Dytiki Ellada	**Norway**	Cataluña	
Aquitaine	Dytiki Makedonia	Agder og Rogaland	Comunidad de Madrid	
Auvergne	Ionia Nisia	Hedmark og Oppland	Comunidad Foral de Navarra	
Basse‐Normandie	Ipeiros	Nord‐Norge	Comunidad Valenciana	
Bourgogne	Kentriki Makedonia	Oslo og Akershus	Extremadura	
Bretagne	Kriti	Sør‐stlandet	Galicia	
Centre (FR)	Notio Aigaio	Trøndelag	Illes Balears	
Champagne‐Ardenne	Peloponnisos	Vestlandet	La Rioja	
Corsica	Sterea Ellada	**Poland**	País Vasco	
Franche‐Comté	Thessalia	Dolnoslaskie	Principado de Asturias	
Haute‐Normandie	Voreio Aigaio	Kujawsko‐Pomorskie	Región de Murcia	
AOle de France				
